# Regulating Cell Apoptosis on Layer-by-Layer Assembled Multilayers of Photosensitizer-Coupled Polypeptides and Gold Nanoparticles

**DOI:** 10.1038/srep26506

**Published:** 2016-05-23

**Authors:** Ruirui Xing, Tifeng Jiao, Kai Ma, Guanghui Ma, Helmuth Möhwald, Xuehai Yan

**Affiliations:** 1 State Key Laboratory of Metastable Materials Science and Technology, Yanshan University, Qinhuangdao 066004, P. R. China; 2 Hebei Key Laboratory of Applied Chemistry, School of Environmental and Chemical Engineering, Yanshan University, Qinhuangdao 066004, P. R. China; 3 National Key Laboratory of Biochemical Engineering, Institute of Process Engineering, Chinese Academy of Sciences, Beijing 100190, P. R. China; 4 Max Planck Institute of Colloids and Interfaces, Am Mühlenberg 1, D-14476, Potsdam/Golm, Germany

## Abstract

The design of advanced, nanostructured materials by layer-by-layer (LbL) assembly at the molecular level is of great interest because of the broad application of these materials in the biomedical field especially in regulating cell growth, adhesion, movement, differentiation and detachment. Here, we fabricated functional hybrid multilayer films by LbL assembly of biocompatible photosensitizer-coupled polypeptides and collagen-capped gold nanoparticles. The resulting multilayer film can well accommodate cells for adhesion, growth and proliferation. Most significantly, controlled cell apoptosis (detachment) and patterning of the multilayer film is achieved by a photochemical process yielding reactive oxygen species (ROS). Moreover, the site and shape of apoptotic cells can be controlled easily by adjusting the location and shape of the laser beam. The LbL assembled multilayer film with integration of functions provides an efficient platform for regulating cell growth and apoptosis (detachment).

Targeted cell detachment is of significant interest and presents new capabilities and opportunities in areas such as biomedicine, cell biology, implantable biomaterials, and tissue engineering^1^,^2^,^3^,^4^,^5^,^6^. Among the variety of available techniques for targeted cell detachment (regulation of cell apoptosis)^7^,^8^,^9^,^10^,^11^, the layer-by-layer (LbL) assembly method plays a pioneering role, because it possesses extraordinary advantages for applications of regulating cell growth and apoptosis including easy formation of supported films, flexible control over structures, combination of various ingredients, adjustable physical properties^12^,^13^,^14^,^15^. A wide variety of functional materials such as biological polymers and inorganic nanoparticles can be readily combined for achieving properties corresponding to each type of materials and integrated functionalities, thus leading to the control over mechanical strength and biocompatibility of LbL assembled films^16^,^17^,^18^,^19^. Such adjustable assembled films can be applied for regulating cell growth, differentiation, and apoptosis (detachment). Significantly, construction and activation of the substrate surface of LbL assembled films possibly allows one to manipulate the function of cells at the molecular level^20^.

The cell detachment on films can be regulated through changes of micro-environmental cues between cells and the substrate surface by different methods, such as those electric-current-induced, pH responding^21^,^22^, magnetism induced, temperature-induced^23^,^24^ or UV light-induced^25^,^26^,^27^. Previous studies showed favorable effects on regulating cell apoptosis on multilayers through LbL assembled films, but there are still some disadvantages of these methods. Electricity-induced or UV light-induced cell detachment is particularly attractive for areas of selective cell separation, on-chip patterning and enhanced cell migration, but irreversible alteration of the substrate surface can be caused and the lack of biocompatibility using electric current or UV-light methods can also damage both the multilayers and adherent cells^28^,^29^,^30^. Cells grown on films are sensitive to their physical environment and they can be weeded out on large-scale if the pH or temperature changes. It may thus result in local deviations from the physiological values and eventually be harmful to surrounding cells^31^,^32^.

Based on the above, it still remains a challenge to regulate cell apoptosis (detachment) facilely, reversibly and friendly in terms of the LbL assembly method^33^,^34^,^35^. In this study, reactive oxygen species (ROS)-induced cell apoptosis (detachment) is achieved on LbL assembled multilayers using photosensitizer-coupled polypeptides (5-Mono(4-carboxyphenyl)-10, 15, 20-triphenyl porphine (TPPAc) -poly-L-lysine (PLL)) and gold nanoparticles (AuNPs) stabilized by type I collagen protein (Fig. 1). For regulating cell growth and apoptosis such LbL assembled films show distinct advantages including: i) excellent biocompatibility due to the biocompatible nature of polypeptides and proteins; ii) enhanced mechanical properties due to introduction of gold nanoparticles; and iii) easy control over cell apoptosis and patterning by ROS obtained via photosensitizers with an adjustable laser source. The multilayer films can be readily constructed by alternating adsorption on demand of photosensitizer-coupled polypeptides (TPPAc-PLL) and collagen-conjugated AuNPs on a supported substrate. By this way, the photosensitive molecule (TPPAc) is entrapped in the supporting film, which can be activated with laser illumination of a certain wavelength yielding ROS, responsible for triggering cell apoptosis^36^. Therefore, cells located in the beam of the laser can provoke death, whereas other cells, in contact with the film but not illuminated by the laser, remain intact. LbL assembled multilayers with integration of multiple functions provide an alternative platform for regulating cell behaviors, presenting a potential for further applications in tissue engineering and implantable materials etc.^37^,^38^.

## Results and Discussion

Layer-by-layer (LbL) assembly can be depicted as a versatile method to engineer multifunctional coatings down to the nanometer scale and to fine-tune the entire spectrum of material properties: mechanical, electrical, optical, and biological to achieve a balanced combination specific for the particular application^39^,^40^. In this work, LbL assembly was applied to prepare thin multilayer films for regulating cell apoptosis. To obtain multilayer films with integration of multiple functions such as biocompatibility, enhanced mechanical properties, and photosensitivity (yielding ROS), we first synthesized positively charged photosensitizer-coupled PLL (TPPAc-PLL) and negatively charged collagen-conjugated AuNPs, which are suited for LbL assembly.

**Figure 2:**
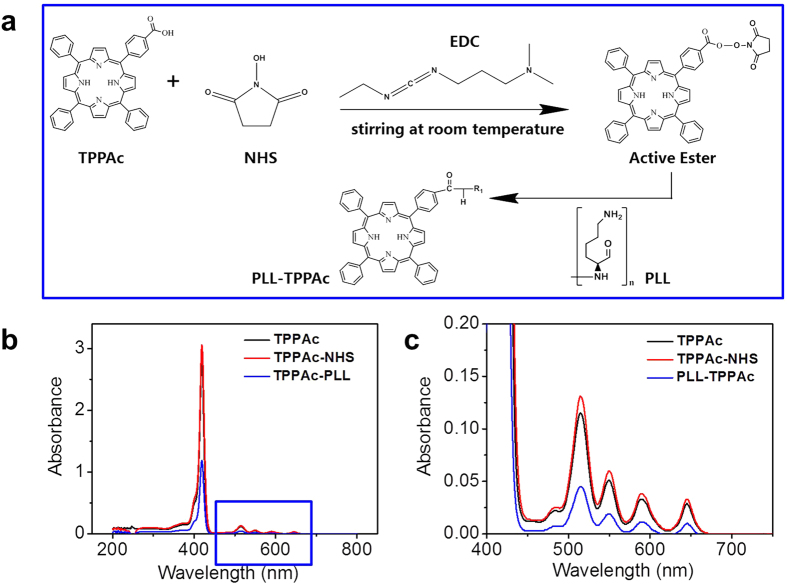
(**a**) Synthesis of the TPPAc-PLL conjugates. (**b**) UV-vis absorption spectra of TPPAc, TPPAc-NHS and TPPAc-PLL water solution. (**c**) Enlarged view of the UV-vis absorption spectrum of the circled part in Fig. 1b.

The positively charged component was synthesized by a standard EDC-NHS reaction including two steps, as shown in Fig. 2a. Due to -Mono(4-carboxyphenyl)-10, 15, 20-triphenyl porphine (TPPAc) having an active carboxyl group, the condensation reaction between COOH group and OH group could easily happen in the presence of 1-ethyl-3-[3-(dimethylamino) propyl] carbodiimide hydrochloride (EDC). Then TPPAc was activated by N-hydroxysuccinimide (NHS) ester to react with polylysine (PLL). The UV absorption spectra of TPPAc, TPPAc-NHS and TPPAc-PLL show the porphin peaks indicatingthat TPPAc was connected to PLL successfully (Fig. 2b,c). All reactions were monitored by thin layer chromatography (TLC) using sorbent technologies with 0.25 mm silica gel plates, which match with the results of UV-vis spectroscopy (Fig. S1). Here, the photosensitizer-coupled polypeptide (TPPAc-PLL) was used as the positively charged component for the LbL assembly.

**Figure 3:**
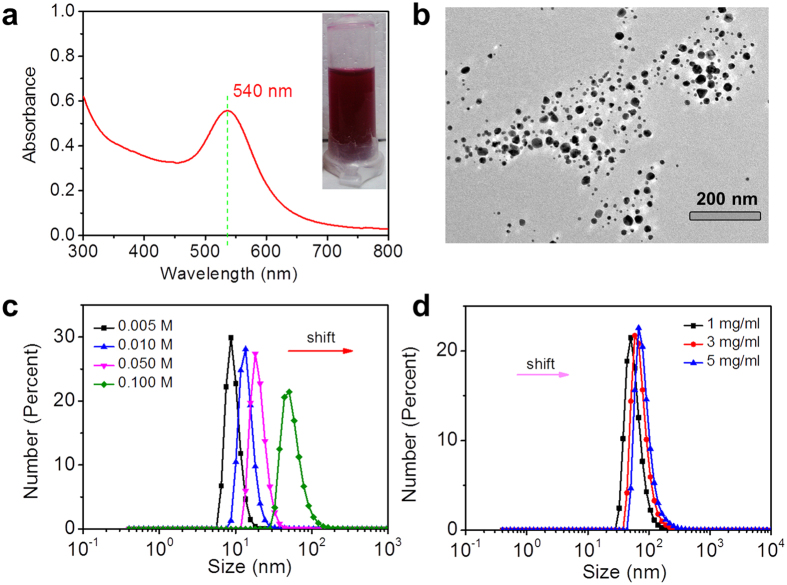
(**a**) UV-vis absorption spectrum of gold-collagen nanoconjugates and photograph of an aqueous solution containing the nanoconjugates (inset). (**b**) TEM images of the nanoconjugates. (**c**) DLS size distribution diagrams of nanoconjugates, which were prepared with the final chloroauric acid concentration 0.005 mM, 0.01 mM, 0.05 mM and 0.1 mM, respectively. (**d**) DLS size distribution diagrams of nanoconjugates prepared with 0.1 mM chloroauric acid concentration and the collagen concentration 1 mg mL^−1^, 3 mg mL^−1^ and 5 mg mL^−1^, respectively.

Our prior work showed, that the negatively charged gold-collagen nanoconjugates can serve as building blocks for preparation of LbL assembled multilayers, and they contribute to the enhancement of mechanical properties and biocompatibility, to promote cell growth and differentiation^41^. Collagen-conjugated AuNPs were rapidly formed, when a 2 mg mL^−1^ acidic collagen aqueous solution was mixed with a 0.1 M chloroauric acid (HAuCl_4_) solution at a temperature of 65 °C with continuous stirring. The UV-vis absorption spectrum of the product shows an absorption band at 540 nm (Fig. 3a), indicating the formation of AuNPs. The color of the mixed solution changes over time and finally changes to red (Fig. 3a inset), further verifying the formation of AuNPs. Besides, the transmission electron microscopy (TEM) image confirms the presence of homogeneously dispersed AuNPs with a size distribution of 20–100 nm (Fig. 3b). Intriguingly, the size of the AuNPs can be controlled flexibly by altering the concentration of HAuCl_4_ or collagen. Clearly, the size of the nanoconjugates analyzed by dynamic light scattering (DLS) demonstrate obvious red-shifts with the amount increase of HAuCl_4_ and the collagen concentration (Fig. 3c,d), implying the formation of larger particles. Compared with the collagen concentration, the final concentration of HAuCl_4_ plays leading roles in regulation of size distribution. Based on the redox mechanism for formation of AuNPs by collagen which was provided in our previous work^41^,^42^,^43^, it can be explained as follows: the smooth supply of raw material ensured the growth of gold nanoclusters, the collagen acting as both reducing agent and stabilizing agent guaranteed the redox reaction and prevented aggregation.

**Figure 4:**
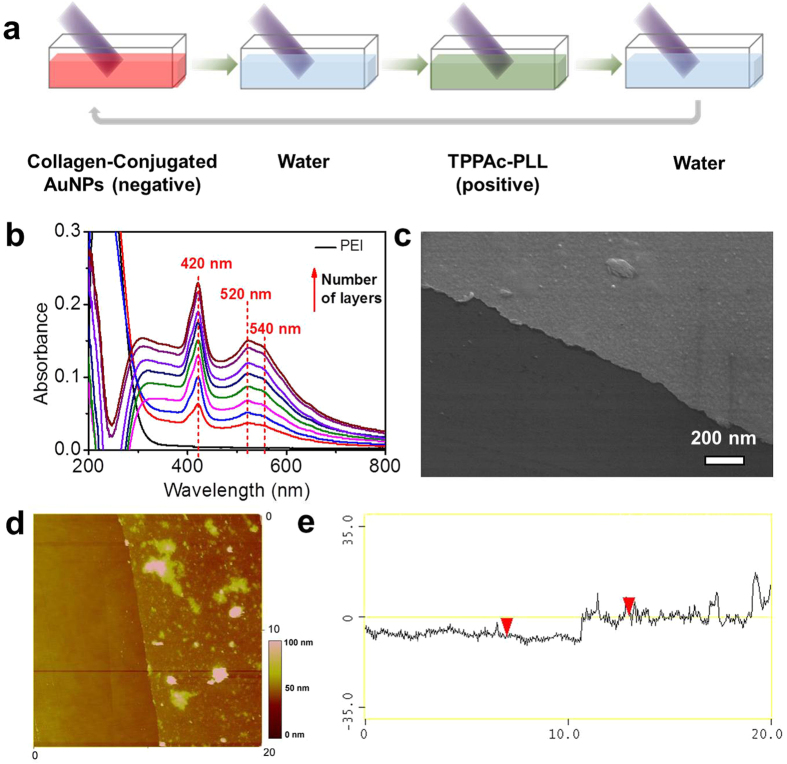
(**a**) Scheme of the LbL film-deposition. Positively charged PEI deposited on the glass slide treated with Piranha solution ahead of schedule. Steps 1 and 3 represent the adsorption of negatively charged colloidal gold-collagen nanoconjugates and positively charged TPPAc-PLL, steps 2 and 4 are washing steps. (**b**) UV-vis absorption spectra of the gold-collagen nanoconjugate/TPPAc-PLL films assembled on a glass slide with increasing number of deposition cycles. SEM (**c**) and AFM (**d**) images showing the surface morphology of a (gold-collagen nanoconjugates/TPPAc-PLL)_5_ film. (**e**) AFM images with section analysis of the (gold-collagen nanoconjugates/PLL-TPPAc)_5_ film, indicating a film thickness of 9.8 nm.

Layer-by-layer assembly here is based on the electrostatic force between collagen-conjugated AuNPs, photosensitizer-coupled polypeptides (TPPAc-PLL) and a glass slide pretreated with a polyetherimide (PEI) monolayer (Fig. 4a). The zeta potential of collagen-conjugated AuNPs and TPPAc-PLL (Fig. S2) was pre-measured to guarantee the applicability for LbL. The two kinds of building blocks were successfully spread on the substrate by such a strategy. This is confirmed by the presence of an absorption band at 540 nm corresponding to the surface plasmon resonance (SPR) absorption of AuNPs, and the two absorption peaks at 420 nm and 520 nm that corresponds to the absorption of TPPAc (Fig. 4b).

The LbL multilayers can be assembled with controlled, variable thickness for generating smart and biocompatible coatings. The cross sectional scanning electron microscope (SEM) image for a film obtained after five deposition cycles shows an even and flat surface with an apparent thickness of 10 nm (Fig. 4c). The AFM image shows that the film has a thickness of approximately 10 nm, and the film surface is covered with evenly distributed AuNPs (Fig. 4d,e). The introduction of inorganic metal cations may improve the mechanical properties of the assembled film to be favorable for cell adhesion and growth^44^. Cell viability measurements were carried out by using the MTT assay of 3T3 fibroblasts after 24 h incubation in the presence of different concentrations of collagen-conjugated AuNPs. The results show, that non-toxicity for AuNPs was established for the cell-cultured lines employed here (Fig. S3). The toxicity of the film is an important point related with cell adhesion and growth and further application in targeted cell detachment.

The reactive oxygen species (ROS) are several highly oxidative molecules and radicals which function as physiological regulators of intracellular signal pathways and also are cytotoxic, whereby they induce cell death, either by apoptosis or by necrosis through oxidation^45^. No obvious cell apoptosis was observed when the cells grew on the (gold-collagen nanoconjugates/PLL)_5_ LbL film illuminated by 559 nm laser irradiation for 10 min (Fig. S4). In such conditions, the AuNPs contribute to neither enough thermal energy nor ROS generation (Fig. S5), thus disenabling the cell death. Here, a photochemical mechanism of cell apoptosis is assigned to the production of ROS under illumination of TPPAc by appropriate laser light. The ROS productivity was investigated using a ROS sensing dye, anthracence-9,10-dipropionic acid (ADPA), under oxidative quenching by the produced ROS^46^. The ADPA reacts with ^1^O_2_ to form an endoperoxide (Fig. S6) and the reduction of ADPA can be followed by the decrease of UV absorption at peaks ranging from 350 to 400 nm. Generally, AuNPs may cause relaxation of an excited state, and thus reduce ROS yielding. However, the ROS yielding was rarely influenced in our case. This may contribute to the two following aspects: i) the content of AuNPs (initial concentration is 10^−4^ mM) incorporated within the film is not high enough; ii) the AuNPs are most likely encapsulated with collagen shells^41^. Both reduce the probability of AuNPs directly interacting with the TPPAc, explaining why the queching is rather low.

**Figure 5:**
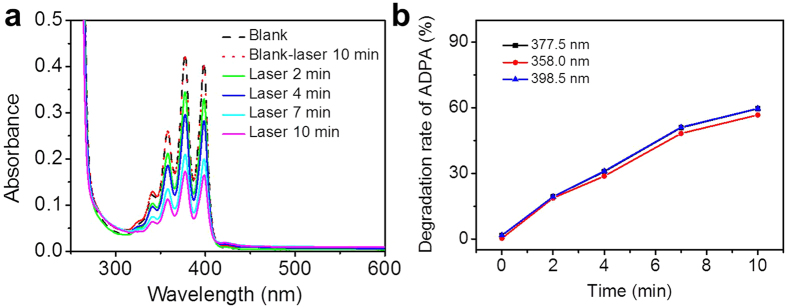
(**a**) Absorption spectra of ADPA in the TPPAc based film system irradiated for 0, 2, 4, 7, and 10 min. (**b**) Photosensitized ADPA bleaching of TPPAc by measuring the absorbance decrease at 377.5nm, 358 nm and 398.5 nm as a function of irradiation time.

The results obtained for irradiation of the TPPAc based film with a light intensity of 40%, 4.0 μs/pixel for 10 min at 559 nm, are presented in Fig. 5a. The degradation of ADPA can be calculated from the UV-vis intensity, assuming a linear dependence, and this is presented in Fig. 5b for the irradiation over time. This ADPA is thermally stable when it is irradiated at 559 nm for 10 min. The UV-vis spectrum hardly shifted before and after illumination, but the intensity of ADPA continuously decreased as the mixtures of ADPA with TPPAc-based film were irradiated at 559 nm within ten minutes. The decrease in intensity indicated the production of ^1^O_2_, which irreversibly reacted with ADPA.

**Figure 6:**
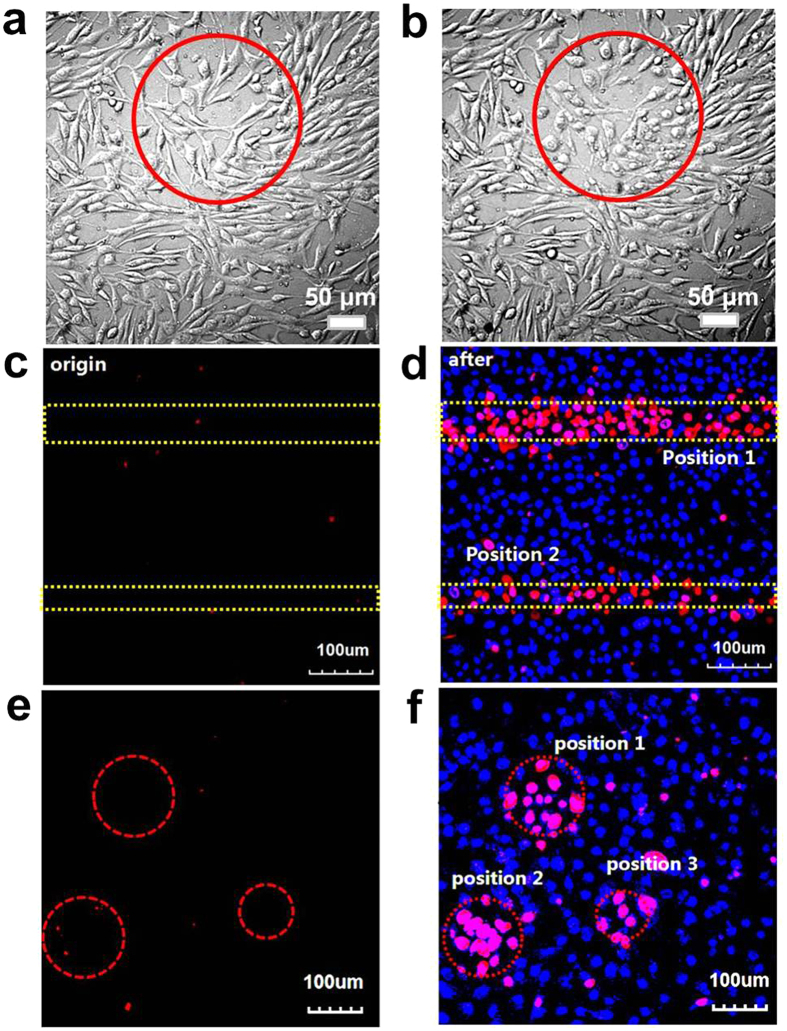
Laser-induced cell detachment. Confocal micrograph of mouse fibroblast 3T3 cells growing on a (gold-collagen nanoconjugates/TPPAc-PLL)_5_ film (**a**), after irradiation with a laser (559 nm, 10 min, light intensity of 40%, 4.0 μs/Pixel) (**b**). Fluorescence micrographs of cells before (**c**,**e**) and after laser irradiation (559 nm, 10 min, light intensity of 40%, 4.0 μs/Pixel) (**d,f** ). The cells were stained with Hoechst 33342 (staining cell nuclei) and propidium iodide (PI, staining nuclei of dead cell). The regions marked by the red circles and yellow dashed lines are exposed to the laser irradiation.

Upon light irradiation, the LbL assembled film is able to generate sufficient ^1^O_2_, that can diffuse out of the matrix, reach the adjacent cell membranes and kill the cells. Confocal microscopy showed that mouse fibroblast 3T3 cells were well adhered and spread throughout the assembled film (Fig. 6a), indicating excellent growth and proliferation prior to light irradiation. After laser irradiation, apparently, cells shrunk and turned round, arranged loosely and at last some cells were floated in the nutrient medium (Fig. 6b). The production of ROS caused by laser irradiation led to a large area of cell damage. However, local illumination induces the death of only those cells that are located in the beam of light; whereas other cells, in contact with the TPPAc based film but not illuminated by the laser, remain intact. This means also the ROS are not moving laterally in the film.

Cell apoptosis can be observed more clearly, as the cells were stained with Hoechst 33342 (staining cell nuclei) and propidium iodide (PI, staining nuclei of dead cell). Before light irradiation, all cells in Fig. 6c,e were rarely stained by PI showing red fluorescence, which verified the cell viability. Concomitant with illumination, the nuclei of the compromised cells were observed to increase theincorporation of PI, as indicated by the red fluorescence (Fig. 6d,f). The site and shape of cell apoptosis on the LbL assembled multilayers can be controlled easily by adjusting the location and shape of the laser beam. So, a photochemically targeted cell apoptosis is realized successfully.

In summary, we have successfully constructed hybrid multilayer films with biocompatibility, enhanced mechanical property and reactive oxygen species (ROS), making use of layer-by-layer (LbL) assembly of photosensitizer-coupled polypeptides and collagen-stabilized gold nanoparticles. Such LbL assembled multilayer films can effectively yield ROS when subject to irradiation with a certain wavelength, thus realizing targeted cell apoptosis (detachment) and cell patterning. The cells remain intact, grow well and proliferate on the assembled multilayer film in absence of exposure to the laser beam. These observations reveal that the strategy, based on LbL assembled multilayers of photosensitive polypeptides and gold nanoparticles, is efficient and flexible for regulating cell growth and apoptosis. It can be readily envisioned that such an assembly approach could be used to assemble other effective constituents for a broader range of application in cell growth, adhesion, differentiation, movement and detachment. Therefore, it may present a promising future for applications in biomedicine, cell biology, medical implantable materials, and tissue engineering.

## Methods

The experimental used materials, Collagen (from calf skin, Type I collagen), gold(III) chloride hydrate (HAuCl_4_), poly-L-lysine hydrobromide (PLL), anthracence-9, 10-dipropionic acid (ADPA), 1-ethyl-3-[3-(dimethylamino) propyl] carbodiimide hydrochloride (EDC), 5-Mono(4-carboxyphenyl)-10, 15, 20-triphenyl porphine (TPPAc), bisBenzimide H 33342 trihydrochloride (Hoechst 33342), propidium iodide (PI) were purchased from Sigma-Aldrich. N-hydroxysuccinimide (NHS) and polyethylenimine (PEI, Mr = 60000) were supplied by Acros Chemicals. Dulbecco’s modled Eagle’s medium (DMEM) and fetal bovine serum (FBS) were purchased from Invitrogen. Dulbecco’s phosphate buffered saline (PBS), trypsin-EDTA (0.5% trypsin, 5.3 mM EDTA tetra-sodium), and the antibiotic agents penicillin-streptomycin (100 U mL^−1^) were supplied by M&C Gene Technology Ltd (Beijing, China). Acetic acid (AA), CH_3_CH_2_OH, sodium chloride (NaCl), sodium hydroxide (NaOH), dimethyl formamide (DMF) and dimethylsulfoxide (DMSO) were products of Beijing Chemicals. Water was prepared in a double-stage Milipore Milli-Q Plus purification system.

The photosensitizer-coupled polypeptide poly-L-lysine-TPPAc (TPPAc-PLL) conjugate was synthesized by a standard EDC-NHS reaction^47^. 5-Mono(4-carboxyphenyl)-10, 15, 20-triphenyl porphine (TPPAc) powder (0.5 mg) was dissolved in DMSO (250 μL), then gently stirred for 5 min at room temperature to get 2 mg mL^−1^ TPPAc solution. Then, 100 μL solution of 1.75 mg mL^−1^ N-hydroxysuccinimide (NHS) in dimethyl formamide (DMF) was mixed with TPPAc solution. After stirring for 5min, 100 μL 2.9 mg mL^−1^ N-(3-Dimethylaminopropyl)-N’-ethylcarbodiimide hydrochloride (EDC) of DMF solution was followed to add to the mixed solution. Then the system was allowed to react for another 4 h. Applying vacuum-rotary evaporation, the active ester powder was obtained by column chromatography, condensation and drying.

The prepared active ester (300 μL, 10 mg mL^−1^, dissolved in DMSO) was added to poly-L-lysine (PLL) (1.2 mL, 10 mg mL^−1^, dissolved in DMSO with the help of a minimum supply of water). The mixture was reacted overnight at room temperature, in turn, unreacted chemicals were removed by dialyzing (MWCO of 3.5 kDa) against DMSO, mixed solution (H_2_O:CH_3_CH_2_OH = 1:1, containing 1% hydrochloric acid), water, phosphate-buffered saline (PBS).

The collagen powder was dissolved in 0.2 M acetic acid (AA) with the help of magnetic stirring and ultrasound to prepare the 2 mg mL^−1^ collagen solution. Subsequently, the pH of the collagen solution was adjusted to 6–6.5 by careful addition of NaOH solution (0.1 M). An amount of 10 μL of HAuCl_4_ (0.01 M) solution was added to the above collagen solution. The mixed solution was incubated for 4 h at a temperature of 65 °C with stirring for the formation of AuNPs. The products were repeatedly washed in high-purity water to remove unnecessary elements for further structural analyses and characterization by dialysis (MWCO of 1.0 kDa).

The formation of AuNPs was monitored by UV-vis spectroscopy with an UV-vis spectrophotometer (UV-2600 of Shimadzu). The size distribution of AuNPs was recorded using a Zetasizer Nano ZS (Malvern Instruments). The surface microstructures of the materials were analyzed by a JEM 1011 transmission electron microscope (TEM) operated at 120 kV.

Film assembly was initially carried out on microscope glass slides cleaned in piranha solution overnight and then thoroughly rinsed with deionized water prior to the use. The LbL coating involved several standard microfabrication procedures^48^. Briefly, a glass slide was immersed in a poly(ether imide) (PEI) aqueous solution (5 mg mL^−1^, containing 0.5% NaCl) for 30 min, rinsed with Milli-Q water for 1 min, dried, and then immersed in gold-collagen nanoconjugate solution for 30 min, rinsed for 1 min, dried again, and then moved to the TPPAc-PLL solution for another 30 min and rinsed with Milli-Q water. The procedure was then repeated with gold-collagen nanoconjugate and TPPAc-PLL, and the LbL assembled film was finally obtained on demand. In order to determine the topography and morphology of the assembled films, atomic force microscopy (AFM) and S-4800 scanning electron microscopy (SEM) were used.

The reactive oxygen species (ROS) produced in the layer by layer film was detected by using 9, 10-dipropionic acid disodium salt (ADPA). The less water-soluble ADPA may take too much time to completely dissolve in PBS, so a saturated solution of ADPA was prepared. The glass slide was soaked in 2 mL ADPA solution, then the film was irradiated for 10 min at 635 nm, under constant stirring and temperature (25 ^o^C). 1 mL of illuminated ADPA solution was monitored by UV-vis spectroscopy with an UV-vis spectrophotometer (UV-2600 of Shimadzu).

For the experiment a mouse embryonic fibroblast cell line, NIH-3T3, was chosen. Cells were cultured in Dulbecco’s modled Eagle’s medium (DMEM) containing 10% bovine serum, 1% penicillin and 1% streptomycin at 37 ^o^C in a 5% CO_2_ environment. The growth of cells was monitored by fluorescence microscopy and with an Olympus confocal microscope system FV 1000 (Olympus, Japan). Laser activation of TPPAc-PLL-based surfaces was conducted using a laser operating at 559 nm of the Olympus confocal microscope system FV 1000.

## Additional Information

**How to cite this article**: Xing, R. *et al.* Regulating Cell Apoptosis on Layer-by-Layer Assembled Multilayers of Photosensitizer-Coupled Polypeptides and Gold Nanoparticles. *Sci. Rep.* **6**, 26506; doi: 10.1038/srep26506 (2016).

## Supplementary Material

Supporting Information

## Figures and Tables

**Figure 1:**
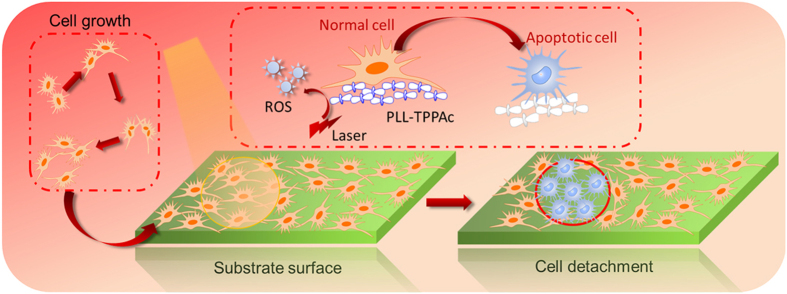
Schematic illustration of reaction oxygen species induced cell apoptosis (detachment) on the layer-by-layer assembled multilayer film.
