# Supplementary material for: Regulating Cell Apoptosis on Layer-by-Layer Assembled Multilayers of Photosensitizer-Coupled Polypeptides and Gold Nanoparticles
Source: Sci Rep. 2016 May 23;6:26506. doi: 10.1038/srep26506 (PMC4876451; doi:10.1038/srep26506)
Supplement: Supporting Information [file srep26506-s1.pdf]

Supporting information

Regulating Cell Apoptosis on Layer-by-Layer Assembled Multilayers of Photosensitizer-Coupled Polypeptides and Gold Nanoparticles

Ruirui Xing[1,2,3], Tifeng Jiao[1,2,*], Kai Ma[2,3], Guanghui Ma[3], Helmuth Möhwald[4], Xuehai Yan[3,*]

[1] State Key Laboratory of Metastable Materials Science and Technology, Yanshan University, Qinhuangdao 066004, P. R. China

[2] Hebei Key Laboratory of Applied Chemistry, School of Environmental and Chemical Engineering, Yanshan University, Qinhuangdao 066004, P. R. China

[3] National Key Laboratory of Biochemical Engineering, Institute of Process Engineering, Chinese Academy of Sciences, Beijing 100190, P. R. China

[4] Max Planck Institute of Colloids and Interfaces, Am Mühlenberg 1, D-14476, Potsdam/Golm, Germany

Corresponding author: Tel.: +86 10 82545004; fax: +86 10 82545024. E-mail addresses: tfjiao@ysu.edu.cn (T. Jiao); yanxh@ipe.ac.cn (X. Yan).

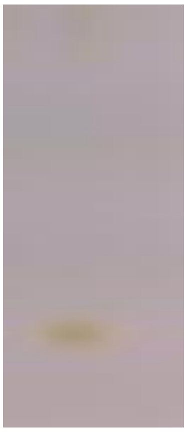

Figure S1. Thin layer chromatography (TLC) of PLL-TPPAc.

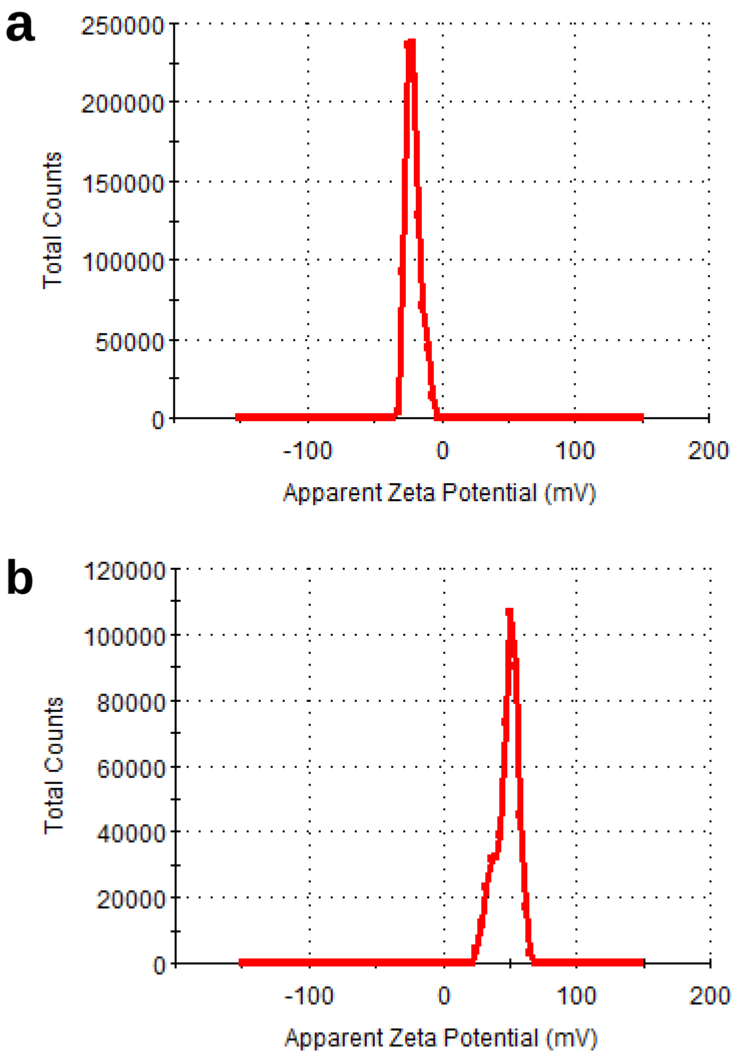

Figure S2. Zeta potential of Collagen-conjugated AuNPs (a) and PLL-TPPAc (b).

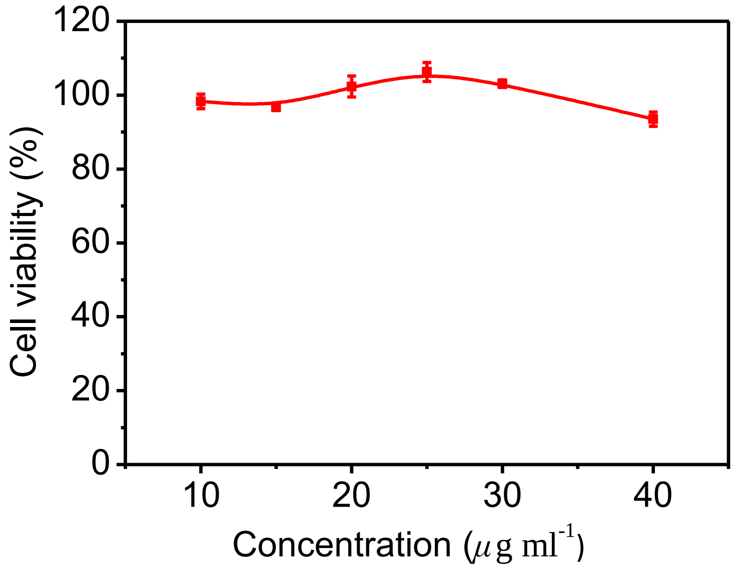

Figure S3. Cell viability measurements carried out by using the MTT assay of 3T3 fibroblasts after 24 h incubation in the presence of different concentrations of collagen-conjugated AuNPs.

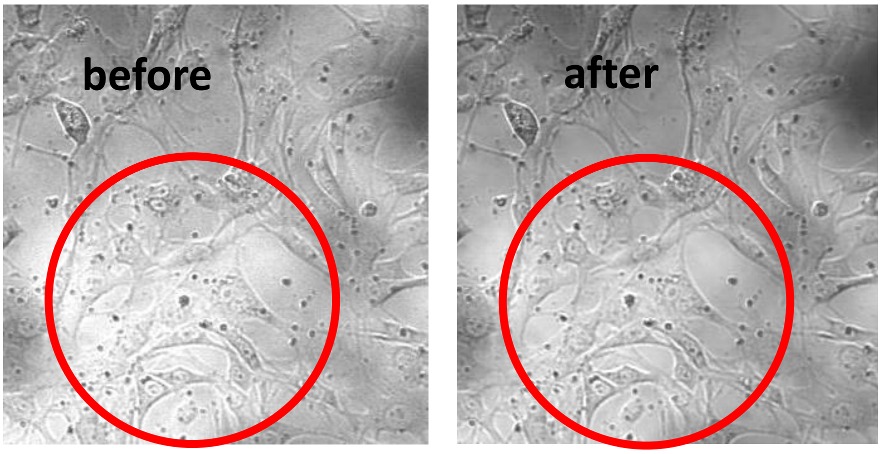

Figure S4. Confocal microscope images of the cells which grow on the (gold-collagen nanoconjugates/PLL)$_5$ LBL film before and after laser irradiation. (laser: 559 nm, 10 min, light intensity of 40%, 4.0 μs/Pixel)

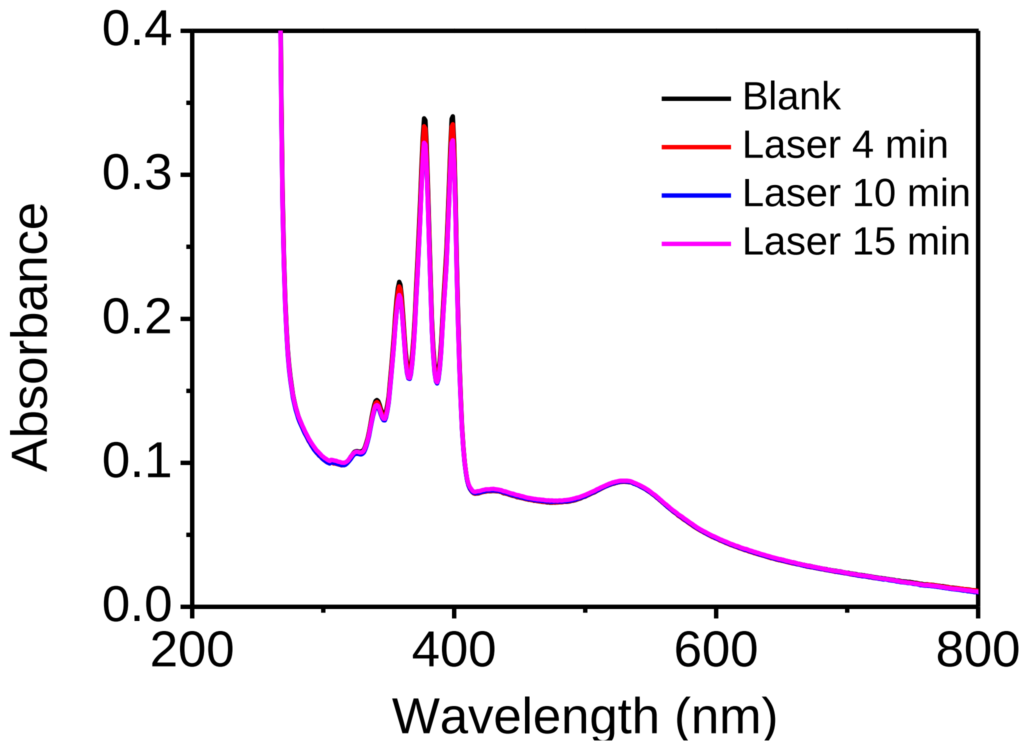

Figure S5. UV-Vis absorption spectra of ADPA in the AuNPs-based film irradiated for 0, 4, 10 and 15 min, showing negligible changes in the intensity, indicating no ROS yielding by the collagen-conjugated AuNPs. Note: The peaks at 377.5 nm, 358 nm and 398.5 nm are the characteristic absorbance of ADPA.

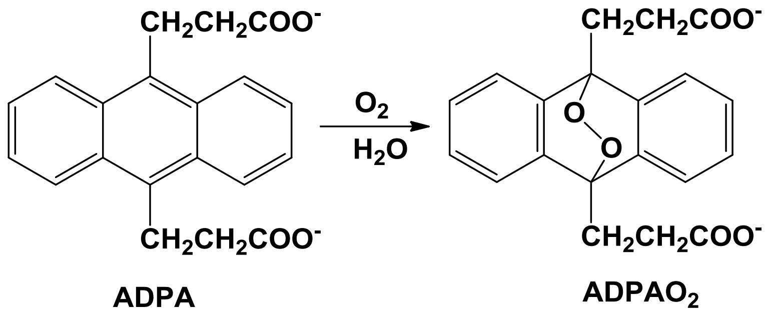

Figure S6. Reaction of ADPA with $^{1}O_2$ to form the endoperoxide $ADPAO_2$.
